# Ontogenetic shifts in wood anatomy and leaf traits in tropical dry forests

**DOI:** 10.1111/nph.70725

**Published:** 2025-11-10

**Authors:** Peter J. Williams, Elise F. Zipkin, Andrés González‐Melo, Beatriz Salgado‐Negret, Roy González‐M., Natalia Norden, Juan Pablo Benavides‐Tocarruncho, Juan Manuel Cely, Julio Abad Ferrer, Daniel García‐Villalobos, Fabián Garzón, Álvaro Idárraga‐Piedrahita, René López‐Camacho, Esteban Moreno, Jhon Nieto, Camila Pizano, Juliana Puentes‐Marín, Nancy Pulido, Katherine Rivera, Felipe Rojas‐Bautista, Viviana Salinas, Juan Felipe Solorzano, María Natalia Umaña

**Affiliations:** ^1^ Ecology, Evolution, and Behavior Program Michigan State University East Lansing MI 48824 USA; ^2^ Department of Integrative Biology Michigan State University East Lansing MI 48824 USA; ^3^ Department of Ecology and Evolutionary Biology University of Michigan Ann Arbor MI 48109 USA; ^4^ Departamento de Biología Universidad Nacional de Colombia Bogotá 111321 Colombia; ^5^ Departamento de Ciencias Forestales Universidad del Tolima Ibagué 730006 Colombia; ^6^ Instituto de Investigación de Recursos Biológicos Alexander von Humboldt Bogotá 110231 Colombia; ^7^ Dirección Territorial Caribe Parques Nacionales Naturales de Colombia Santa Marta 470004 Colombia; ^8^ Fundación Jardín Botánico de Medellín, Herbario “Joaquín Antonio Uribe” (JAUM) Medellín 050010 Colombia; ^9^ Facultad del Medio Ambiente y Recursos Naturales Universidad Distrital Francisco José de Caldas Bogotá 110321 Colombia; ^10^ Instituto de Hidrología, Meteorología y Estudios Ambientales Bogotá 110911 Colombia; ^11^ Departmento de Biología Universidad Icesi Cali 760031 Colombia; ^12^ Department of Liberal Arts School of the Art Institute of Chicago Chicago IL 60603 USA; ^13^ Instituto de Biología Universidad de Antioquia Medellín 050010 Colombia

**Keywords:** Colombia, functional traits, leaf economic traits, seedlings, specific leaf area, trade‐offs, trees, wood density

## Abstract

- Trees experience changes in environmental conditions and biophysical constraints as they grow that may lead to shifts in functional traits. While ontogenetic shifts in leaf traits are relatively well understood, changes in wood anatomy traits from seedlings to adults are less clear, especially in tropical dry forests where drought strongly influences adult wood traits.
- We collected wood and leaf functional trait data for seedlings and adult trees in four dry forest sites along a rainfall gradient in Colombia. Using a Bayesian framework, we quantified intraspecific trait shifts between seedlings and adults, and we compared functional axes and trait correlations between these stages.
- Leaf traits shifted along a clear functional axis from more acquisitive seedlings to more conservative adults, but changes in vessel traits were more complicated. Vessel diameter increased across ontogeny as plant height increased. Wood anatomy traits were less tightly coupled for seedlings than for adults, such that the hydraulic safety–efficiency trade‐off observed in adults appeared to be weaker.
- Our results indicate that wood anatomy traits do not show coordinated ontogenetic shifts among traits because traits associated with hydraulic safety and efficiency are less integrated in seedlings. In dry forests, hydraulic trade‐offs become stronger as trees grow taller.

## Introduction

As trees grow from seedlings to adults, they experience changes in abiotic conditions and in the way they allocate resources such that functional strategies change over ontogeny (Dayrell *et al*., 2018; Barton, 2024). These changes are most clearly observed in traits associated with the leaf economics spectrum (Wright *et al*., 2004; Reich, 2014), where seedling leaves are more resource‐acquisitive to grow faster in competitive but shaded environments, while adult leaves are more conservative to cope with high light yet stressful environments associated with high irradiation (Fortunel *et al.*, 2020; Barton, 2024). In less light‐limited environments, shifts in relative growth rate can still result in leaf shifts from acquisitive seedlings to conservative adults (Dayrell *et al*., 2018; Barton, 2024). Consistent declines in specific leaf area (SLA) through ontogeny have been observed in many species, including both woody and herbaceous plants, and across ecosystems, including tropical forests, grasslands, and coastal dunes (Dayrell *et al*., 2018; Barton, 2024). As with leaf traits, wood traits also change over ontogeny. As trees mature from fast‐growing resource‐acquisitive seedlings to slow‐growing resource‐conservative adults, they often produce denser wood (Hietz *et al*., 2013; Plourde *et al*., 2015). Not all species exhibit increasing wood density with age, though, especially slower‐growing shade‐tolerant species (Rungwattana & Hietz, 2018; González‐Melo *et al*., 2022). Mechanical and developmental constraints also influence ontogenetic differences in wood traits. Large trees must support greater weight and resist higher forces caused by factors such as wind (Rowe & Speck, 2005), so trees may increase fiber wall thickness as they grow to add mechanical strength (Rungwattana & Hietz, 2018). Large trees must also transport water further distances up through the xylem without breaking the water column, which requires wider vessels (Koch *et al*., 2004). Vessel diameter increases predictably with stem length across hundreds of plant species (Olson *et al*., 2014, 2018), so seedlings may be incapable of producing vessels as wide as adults due to their smaller height. While it is well established that plants undergo substantial morphological shifts over ontogeny, a comprehensive examination of how these changes reflect shifts in ecological strategies across different functions – such as water transport and carbon uptake – and across a broad range of species has been lacking.

In tropical dry forests, drought plays a profound role in determining the traits of adult trees (Murphy & Lugo, 1986; González‐M. *et al*., 2021), but seedlings face different local environmental conditions than adult trees such that functional traits may differ from adults. Seedlings growing in shaded forest understories experience lower evaporative demand than adults, potentially leading to reduced water stress (Niinemets, 2010). Although, dry forests with many deciduous species do not provide shaded canopies in times of drought, and increased litterfall from deciduous adults may cause additional stress for seedlings during droughts (Wang *et al*., 2022). Compared to seedlings, adults have deeper root systems to access deep water sources as well as greater carbon storage pools, potentially making seedlings more vulnerable to droughts (Niinemets, 2010). Conversely, seedlings are less vulnerable to embolism because they have narrower vessels (Olson *et al*., 2014, 2018; McCulloh *et al*., 2015) and shorter distances for water transport (Zhang *et al*., 2009) compared to adults. Given these complex differences in water availability and use between life stages, it is unclear how traits might shift from seedlings to adults in tropical dry forests. In particular, while ontogenetic shifts in traits such as SLA and vessel diameter have been well studied in other species and ecosystems, it remains unclear how other wood anatomy traits differ between stages.

If ontogenetic trait shifts are determined by differences in water and light environments between stages, then the magnitude of trait shifts may vary by site depending on abiotic conditions. Light availability is often a major limiting factor for seedlings (Augspurger, 1984; Kitajima, 1994). Thus, wetter forests with denser canopies and lower light availability in the understory may impose stronger contrasts between environments experienced by seedlings compared to adults. Seedlings in these forests benefit from reduced water stress due to the more humid understory, but they grow under shaded conditions (Tripathi *et al*., 2020). By contrast, adult trees in the canopy are exposed to full sunlight and higher evaporative demand, creating a sharp ontogenetic gradient for both light and water availability. Moreover, drier forests, which are generally more open and dominated by deciduous species, tend to exhibit smaller differences in microenvironmental conditions between understory and canopy. Therefore, the environmental contrast between the understory and the canopy, and thus between seedlings and adults, should be less pronounced in drier forests. Thus, ontogenetic trait shifts may be less pronounced in drier forests and more pronounced in wetter forests.

The magnitude of ontogenetic shifts likely varies substantially among species. For example, species with longer leaf lifespans at maturity may have greater ontogenetic shifts in leaf traits as they shift along the leaf economics spectrum from a resource acquisition strategy in the seedling stage to a resource conservation strategy in adulthood (Mediavilla *et al*., 2014). Fast‐growing pioneer species have greater shifts in wood density as they transition from acquisitive seedlings to conservative adults, compared to slow‐growing shade‐tolerant species that are already conservative as seedlings (González‐Melo *et al*., 2022). Ontogenetic trait shifts may also vary depending on leaf phenology or growth form. Deciduous species, which can avoid drought by dropping their leaves, tend to have more acquisitive and more hydraulically efficient traits compared to evergreen species (Ribeiro *et al*., 2022). Compared to trees, lianas tend to have more acquisitive traits (at least in dry forests) and different xylem traits (Dias *et al*., 2019; Medina‐Vega *et al*., 2021). While the traits of deciduous species and lianas differ from evergreen trees, it is unclear whether the magnitude of ontogenetic trait shifts also differs, which would depend on whether the traits of these groups become more or less differentiated over ontogeny.

Beyond shifts in individual traits, it is unclear whether traits in seedlings are coordinated along the same functional axes as adults. For adult trees in tropical dry forests, wood and leaf traits form two main orthogonal axes. Along the first axis, traits such as hydraulically weighted vessel diameter (d_h_), vessel density (VD), pit diameter aperture (DA_pit_), and hydraulic conductivity represent a trade‐off between hydraulic safety and hydraulic efficiency (González‐M. *et al*., 2021; Umaña *et al*., 2023). At one end of this axis are trees with many narrow vessels and smaller pits, increasing resistance to embolism but transporting water less efficiently. At the other end are trees with few wide vessels that efficiently transport water, promoting faster growth but increasing the risk of drought‐induced embolisms. Along the second axis, traits such as SLA, leaf dry matter content (LDMC), and wood specific gravity (WSG) represent a continuum of tissue investment, akin to the resource acquisitive–conservative trade‐off (González‐M. *et al*., 2021; Umaña *et al*., 2023). At one end of this axis are trees with thin, less dense leaves and less dense wood (‘cheap’ tissues), and at the other end are trees with thick, dense leaves and dense wood (‘costly’ tissues). If the trade‐offs among traits are similar between seedlings and adults, then we would expect coordinated ontogenetic shifts of multiple traits along these functional axes. For example, a decrease in SLA from seedlings to adults would be accompanied by an increase in LDMC and an increase in WSG, representing an ontogenetic shift along the trait investment axis. Likewise, an increase in d_h_ would be accompanied by a decrease in VD and an increase in DA_pit_, representing an ontogenetic shift along the hydraulic efficiency–safety axis. However, it is unclear whether seedlings do, in fact, have the same functional axes as adults as there have been few studies that compare multiple functional traits in seedlings and adults, especially for wood traits.

Given the differences in light, water, and biophysical constraints, traits in seedlings – especially wood traits – may not be coupled in the same way as in adults. In drier places with strong seasonality, such as tropical dry forests, the trade‐off between hydraulic efficiency and safety is strong (Liu *et al*., 2021), but this trade‐off is weak for woody species globally (Gleason *et al*., 2016). In dry forests, seedlings and adults are both constrained by water availability, but seedlings may experience water stress differently because of canopy shading (depending on forest deciduousness), shallower roots, and reduced carbon storage pools. Seedlings are also less vulnerable to embolism than adults due to their shorter stature (Olson *et al*., 2014, 2018; McCulloh *et al*., 2015), so seedlings may not need to be as hydraulically ‘safe’ as adults. However, it is unclear whether seedlings exhibit the same safety–efficiency trade‐off as adults, in which case they would likely fall on the ‘efficiency’ end, or whether hydraulic safety and hydraulic efficiency are decoupled in seedlings. Seedlings in tropical dry forests do exhibit trade‐offs among wood traits, in particular a trade‐off between high parenchyma fraction and high fiber fraction (González‐Melo *et al*., 2025), but it is still unknown whether seedlings have the same trade‐offs for the same sets of traits as adults.

Here, we present data from four tropical dry forests in Colombia to investigate ontogenetic trait shifts between seedlings and adults. We aimed to address the following questions: (1) How do traits shift across ontogeny in tropical dry forests? (2) Does the magnitude of ontogenetic trait shifts vary along a rainfall gradient? (3) Are functional traits in seedlings coordinated along the same functional axes observed in adults? For question 1, we predicted that SLA would decrease and that LDMC and WSG would increase as trees grow from seedlings to adults, in line with observed shifts of increasing tissue investment. We also predicted that TFW would increase as adult trees require greater mechanical strength. We predicted that d_h_ would increase, given that d_h_ is strongly correlated with plant height. Last, given the negative correlation between d_h_ and VD in adults and the expected increase in d_h_, we predicted that VD would decrease. For question 2, we predicted that the magnitude of ontogenetic trait shifts would be greater in wetter sites where the difference in environmental conditions between seedlings and adults is more pronounced. For question 3, we predicted that seedlings and adults would exhibit the same tissue investment axis, comparable to the widely observed leaf economics spectrum. We also predicted that seedlings would exhibit the same hydraulic safety–efficiency axis as adults under the assumption that seedlings and adults face similar hydraulic trade‐offs, though we investigated this assumption.

## Materials and Methods

### Study sites

The study was conducted in four mature tropical dry forests in Colombia that differ in water availability, floristic composition, and forest structure (González‐M. *et al*., 2019, 2021). The mean annual precipitation of the study sites varied from 677 to 1597 mm (Table 1). Sites also varied in the amount of light that penetrates the canopy and the proportion of trees that are deciduous (Table 1), and these factors may influence the local environments experienced by seedlings. Between 2013 and 2014, a 1‐ha permanent plot was established in each forest to monitor the dynamics of all stems with a diameter at breast height (DBH) ≥ 2.5 cm, which we define as adults (González‐M. *et al*., 2021). In 2021, we established one hundred 1‐m^2^ seedling plots within each of the four 1‐ha plots for a total of 400 seedling plots (González‐Melo *et al*., 2025). The seedling plots were systematically located in 10 × 10 m quadrats within each 1‐ha plot. All woody seedlings, which we defined as individuals taller than 20 cm and with a DBH < 1 cm (Comita *et al*., 2023), were tagged, measured, mapped, and identified to species within these plots. At each seedling plot, light intensity was calculated as a gap light index derived from hemispherical photographs using a fisheye lens (see González‐Melo *et al*., 2025), and the mean light intensity across all 100 plots was calculated for each site (Table 1). All seedlings were understory and nonreproductive individuals.

**Table 1:** Study sites, numbered from wettest (1) to driest (4).

Site number	Site name	Latitude	Longitude	Mean annual temperature (°C)	Total annual precipitation (mm)	Aridity index	Mean light intensity (%)	Deciduous basal area (%)
1	Jabirú	5°03′N	74°49′W	27.3	1597	0.90	14	17
2	Colorados	9°56′N	75°06′W	26.2	1381	0.85	7	48
3	Cotové	6°31′N	75°49′W	26.1	1240	0.80	28	45
4	Tayrona	11°18′N	74°07′W	28.2	677	0.30	30	70

Data were obtained from González‐M. *et al*. (2021) and González‐Melo *et al*. (2025). Aridity index was calculated as total annual precipitation divided by potential evapotranspiration, with lower values corresponding to more arid sites. Light intensity was measured in the wet season. Light intensity was measured at 1 m above the ground (see González‐Melo *et al*., 2025 for details).

### Species selection

From the collected seedling data, we selected woody species whose adult stages could potentially be represented in adult surveys. This included trees, lianas, and shrubs but excluded plants such as subshrubs that would never achieve a DBH > 2.5 cm. For species found at multiple sites, we measured traits at each site and treated populations across sites as independent. Therefore, we use ‘populations’ to describe our sample sizes. In total, we had 61 seedling populations (54 species) and 224 adult populations (173 species) with data for at least one trait. Of the 285 total seedling and adult populations, seven seedling and 26 adult species had populations at two sites, eight adult species had populations at three sites, and three adult species had populations at all four sites. To analyze trait shifts for questions 1 and 2, we used 34–45 populations (29–39 species) which had both seedling and adult data for a given trait, though the exact sample size depended on the trait. Of these 34–45 populations, 13–17 were deciduous, 21–28 were evergreen, 6–7 were lianas, 28–38 were free‐standing, and each of the four sites included 6–13 populations, again depending on the trait. To calculate functional trait axes for question 3, we used 37 seedling populations (33 species) and 195 adult populations (184 species) which had complete trait data. For each analysis, we included all species with complete data for the trait or set of traits included in that analysis, meaning that sample sizes differed across analyses. See Supporting Information Table S1 for the sample sizes for each trait.

### Trait data collection

Traits were chosen based on their relevance to plant function, their feasibility for measurement across ontogenetic stages, and their widespread use in ecological trait databases (e.g. TRY) and comparative studies. For wood anatomical traits, we focused on traits that capture two key functions: water transport, which relates to hydraulic efficiency and embolism risk, and biomechanical support. For leaf traits, we selected variables that represent light capture strategies or are components of the leaf economics spectrum, as they are widely used to characterize trade‐offs in resource acquisition and conservation and have been extensively studied both in tropical systems and globally. For seedlings, we identified the most common species within the 1‐m^2^ plots, representing a diverse array of ecological strategies and wood anatomical features. In the areas surrounding each 1‐ha plot, we collected 2–6 seedlings per species, with heights between 20 cm and 130 cm, for trait collection (González‐Melo *et al*., 2025, Table 2). For adults, we measured traits in 1–8 individuals, depending on the abundance of the species in the plot (González‐M. *et al*., 2021). All samples were collected in the wet season.

**Table 2:** Functional traits included in the study.

Trait	Abbreviation	Units	Description
Leaf area	LA	cm^2^	Area displayed for light capture (Poorter & Rozendaal, 2008)
Leaf dry matter content	LDMC	g g^−1^	Part of the leaf economics spectrum, exemplifying the balance between resource acquisition and conservation. Higher LDMC values typically indicate a greater investment in structural material and a strategy for conserving resources (Messier *et al*., 2017)
Specific leaf area	SLA	cm^2^ g^−1^	Part of the leaf economics spectrum. Higher SLA values indicate a strategy of investing less in structural material for a larger leaf area, which can enhance photosynthesis but may also lead to higher water loss (Wright *et al*., 2004)
Wood specific gravity	WSG	[unitless]	Compound trait representing trade‐offs among support, water transport, and storage (Chave *et al*., 2009). WSG (wood density divided by water density) is unitless but is equivalent to wood density with units g cm^−3^ when water is 4°C (i.e. at maximum water density)
Thickness of fiber wall	TFW	μm	Mechanical support, and hydraulic safety. Thicker fiber walls provide increased mechanical strength and are associated with reduced risk of cavitation (Jacobsen *et al*., 2005)
Hydraulically weighted vessel diameter	d_h_	μm	Hydraulic efficiency and safety. Larger vessels (higher d_h_ values) promote higher hydraulic conductance by more efficiently transporting water, but larger vessels may also increase risk of cavitation (Scholz *et al*., 2013; Isasa *et al*., 2023), though d_h_ is not necessarily linked to embolism risk (Hacke *et al*., 2017; Lens *et al*., 2022)
Vessel density	VD	vessels mm^−2^	Hydraulic safety. Higher vessel density is associated with reduced risk of embolism and provides greater redundancy in the event of embolism (Levionnois *et al*., 2021; Ewers *et al*., 2023)
Pit diameter aperture	DA_pit_	μm	Hydraulic efficiency and safety. Smaller pits (lower DA_pit_ values) increase hydraulic resistance but are associated with reduced risk of embolism (Choat *et al*., 2008; Levionnois *et al*., 2021), though pit membrane thickness is a better predictor of embolism risk (Levionnois *et al*., 2022)

We measured three leaf traits (Table 2). For each plant population, either seedling or adult, we collected fully developed and healthy leaves. For adults, only sunny leaves were collected. Leaves were scanned for calculating leaf area (LA) and then dried in an oven for 48 h at 60°C to determine dry mass. SLA was measured as LA over dry mass. LDMC was calculated by dividing leaf dry mass by leaf fresh mass (Cornelissen *et al*., 2003).

We measured five wood traits (Table 2). For each seedling sample, we obtained a 5‐cm stem sample near the base, which was then split into two parts. One part was used to measure WSG, whereas the other was preserved in a glass container with a solution of 96% ethanol and water (50 : 50) for later laboratory processing (González‐Melo *et al*., 2025). For adults, we collected branches from 8 to 10 m above ground, except for understory species, where the highest possible branches were collected. Wood samples were selected from lignified branches measuring 15–23 mm in diameter and 15–20 cm in length. The branches were dried for 48–72 h at a temperature of 60–65°C to prevent warping and cracking. To measure the remaining functional traits, xylem tissue sections (15–40 μm thick) were cut radially, tangentially, and transversely using a Leica RM2255 microtome. Sections were bleached, stained, dehydrated, and cleaned following Gärtner & Schweingruber (2013) and Yeung *et al*. (2015) protocols. We measured VD, hydraulically weighted diameter (d_h_), pit diameter aperture (DA_pit_), and thickness of the fiber wall (TFW) following standard protocols (Scholz *et al*., 2013; Salgado‐Negret *et al*., 2016). It is important to note that the seedling samples were kept hydrated before processing while the adult samples were oven dried. Given that dehydration irreversibly reduces pit membrane thickness (Li *et al*., 2016; Zhang *et al*., 2017; Kotowska *et al*., 2020), our measurements of DA_pit_ may not be comparable between seedlings and adults.

A summary of trait values for seedlings and adults is provided in Table S1.

### Data analysis

We estimated ontogenetic trait shifts (question 1) by assuming that the difference in trait values between adults and seedlings of the same population (i.e. same species and same site) was derived from a normal distribution, and we interpreted trait differences whose 95% credible intervals did not cross zero as significant. We modeled the effect of site on ontogenetic trait shifts (question 2) by including separate intercepts for each site. We then calculated the pairwise differences among site intercepts to determine whether the ontogenetic shifts between each pair of sites significantly differed. Furthermore, to account for additional variation in trait shifts, we modeled the effect of site on ontogenetic trait shifts as above while including additional effects of phenology (deciduous or evergreen) and growth form (liana or free‐standing). Studies have reported differences in traits between deciduous and evergreen species (Ribeiro *et al*., 2022) and between lianas and trees (Dias *et al*., 2019; Medina‐Vega *et al*., 2021), though it is unclear whether these groups differ in the magnitude of ontogenetic trait shifts. For this last model, the change in trait value was assumed to come from a normal distribution with a mean μ, which we estimated as follows:

(Eqn 1) $$\mu_{i}=\alpha_{k}*\mathrm{sit}e_{i,k}+\beta_{1}*{\text{deciduous}}_{i}+\beta_{2}*{\text{liana}}_{i}$$

The parameter *α*_*k*_ is the intercept, which is different across the sites (*k* = 1, 2, 3, or 4), *β*_1_ is the effect of being deciduous, and *β*_2_ is the effect of being a liana. We ran all three models (change in trait only, site effects, site effects plus phenology and growth form effects) for all traits. We included effects of phenology and growth form because deciduous species and lianas may have different traits than evergreen species and trees, and the four sites vary in the proportion of deciduous species and lianas in each sample.

To determine functional trait axes (question 3), we developed a PCA that we implemented using a Bayesian framework. We performed this analysis for adults (*n* = 195 populations), for seedlings (*n* = 37), and for adults and seedlings combined (*n* = 243). See Methods S1 for details on how the Bayesian PCA with varimax rotation was calculated. A key advantage of using Bayesian methods for this analysis is that it allows for the estimation of credible intervals around the loadings, providing a measure of uncertainty and variance around the vectors, which is not possible with classic PCA methods that estimate parameters with frequentist approaches.

We further investigated functional trait axes (question 3) by analyzing the correlation between pairs of traits. We assumed that the standardized trait values for a given pair of traits were derived from a multinormal distribution, and we estimated the correlation coefficient from the resulting covariance matrix. In cases where trait correlation values were similar between adults and seedlings, we ran simple linear models for both adults and seedlings and compared the posterior distributions of the intercepts and the slopes. We selected the trait with the greater difference in seedling and adult values to be the dependent variable, with the other trait as the independent variable.

For all analyses, we used a Bayesian framework to estimate parameters. We ran models using the nimble package v.1.1.0 in R v.4.2.1. For each analysis, we specified vague priors for all parameters and ran three Markov chains for 4000 iterations, discarding the first 1000 iterations as burn‐in. We assessed model convergence by checking trace plots and verifying that Gelman‐Rubin R statistics were < 1.1 (Gelman & Rubin, 1992).

## Results

### Question 1: Ontogenetic trait shifts

**Fig. 1:**
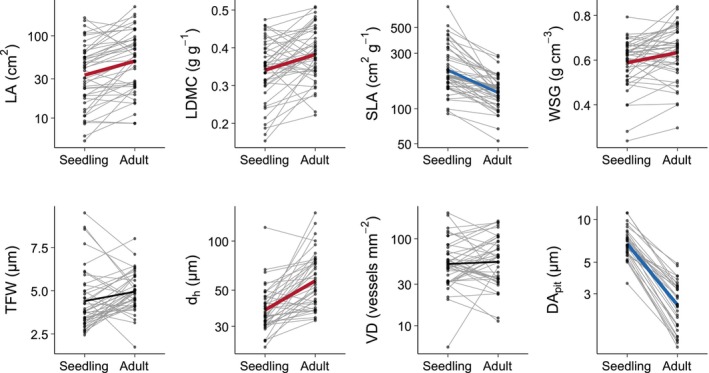
Ontogenetic trait shifts from seedlings to adults. Each line represents a pair of adult and seedling trait values of the same population (same species and site), with overall mean trait values and shifts represented by a bold colored line. Significantly positive mean trait shifts (increasing from seedling to adult) are indicated by red lines, significantly negative mean trait shifts (decreasing from seedling to adult) are indicated by blue lines, and nonsignificant mean trait shifts are shown in black, where trait shifts are significant when the 95% credible interval does not include zero. Decreases in DA_pit_ may be due to methodological differences between adults and seedlings (see the Materials and Methods section). Note that LA, SLA, d_h_, VD, and DA_pit_ are shown on log scales. DA_pit_, pit diameter aperture; d_h_, hydraulically weighted vessel diameter; LA, leaf area; LDMC, leaf dry matter content; SLA, specific leaf area; TFW, thickness of fiber wall; VD, vessel density; WSG, wood specific gravity.

Most traits showed significant ontogenetic shifts across species. Compared to seedlings from the same populations, trait values of adults increased for LA (mean shift in standardized trait value = 0.41, 95% CI: [0.24, 0.58]), LDMC (0.48 [0.22, 0.75]), and WSG (0.33 [0.13, 0.53]; Fig. 1). By contrast, SLA decreased (SLA; −1.03 [−1.27, −0.79]). For wood anatomy traits, trait values significantly increased for vessel diameter (d_h_; 1.01 [0.71, 1.31]) but did not change for VD (0.09 [−0.24, 0.42]) or thickness of fiber wall (TFW; 0.36 [−0.00, 0.70]). Pit diameter aperture greatly decreased across all species (DA_pit_; −2.03 [−2.22, −1.82]), though this may be attributed to methodological differences between adults and seedlings (see the Materials and Methods section).

### Question 2: Ontogenetic shifts across a rainfall gradient

**Fig. 2:**
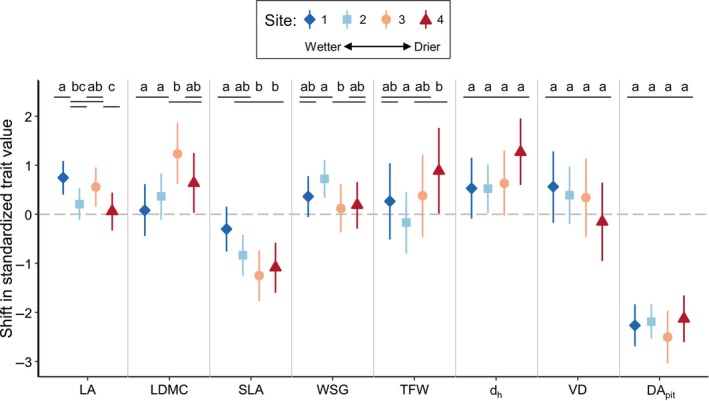
Differences in ontogenetic trait shifts across sites. Positive values indicate an increase in trait value from the seedling stage to the adult stage, whereas negative values indicate a decrease in trait value. Colored shapes represent mean estimated trait shifts for a given site, and colored lines represent 95% credible intervals. For a given trait, sites with significantly different trait shifts are indicated by different letters and nonoverlapping lines at the top of the figure. Differences between sites are significant when the 95% credible interval for the difference between site intercepts does not include zero. The model used to estimate these results includes effects of phenology (deciduous vs evergreen) and growth form (liana vs free‐standing) on trait shifts. DA_pit_, pit diameter aperture; d_h_, hydraulically weighted vessel diameter; LA, leaf area; LDMC, leaf dry matter content; SLA, specific leaf area; TFW, thickness of fiber wall; VD, vessel density; WSG, wood specific gravity.

The magnitude of ontogenetic shifts for leaf traits varied among sites, though not strictly along the rainfall gradient (Fig. 2). In the wettest site (site 1), SLA decreased from the seedling stage to the adult stage less steeply than in the two driest sites (sites 3 and 4). Sites 1 and 2 had a smaller shift in LDMC than site 3. Site 1 had a greater shift in LA than sites 2 and 4, while site 3 also had a greater shift in LA than site 4. For wood anatomy traits, the strength of ontogenetic shifts differed little across sites with contrasting environmental conditions. We did not find significant differences in the strength of ontogenetic changes among sites for d_h_, VD, or DA_pit_. The shift in d_h_ was greater in the driest site (site 4) than in the two wettest sites (sites 1 and 2) only when the effects of leaf phenology and growth form were not accounted for (Fig. S1). The shift in TFW was greater in site 4, where TFW increased, than in site 2, where TFW decreased. The shift in WSG was greater in site 2 than in site 3.

Leaf phenology and growth form also explained some of the variation in the magnitude of ontogenetic shifts (Fig. S2). SLA decreased from the seedling to adult stage overall, but the decrease in SLA was steeper for deciduous species than for evergreen species (mean effect of leaf phenology [deciduous] on shift in SLA = −0.710, 95% CI [−1.167, −0.254]) and tended to be less steep for lianas than for free‐standing species (mean effect of growth form [liana] on shift in SLA = 0.567, 95% CI [−0.037, 1.184], Figs S2, S3). The increase in d_h_ from seedlings to adults tended to be steeper for lianas compared to free‐standing species (mean effect of growth form [liana] on shift in d_h_ = 0.728 [−0.031, 1.499], Figs S2, S3), though this effect was not significant. Shifts in VD tended to be more negative for deciduous species than for evergreen species (mean effect of leaf phenology [deciduous] on shift in VD = −0.654 [−1.361, 0.036]), but neither group had consistent ontogenetic shifts in VD in either direction (Figs S2, S3).

### Question 3: Coordination of functional traits for adults and seedlings

**Fig. 3:**
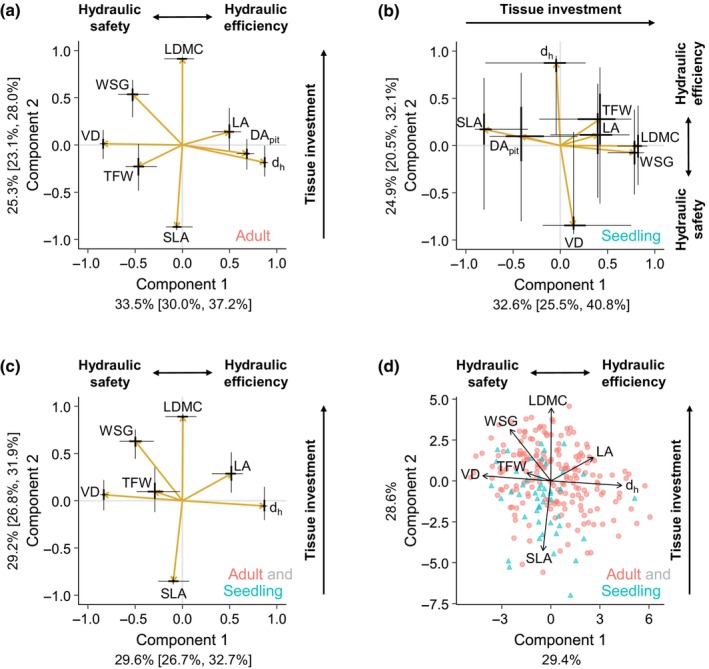
Functional trait PCA with varimax rotation for (a) adults, (b) seedlings, and (c) adults and seedlings combined. Gold arrows represent median loadings, with 50% credible intervals (thick lines) and 95% credible intervals (thin lines) for loadings. The median variance explained by each component is shown with 95% credible intervals in brackets. (d) For adults and seedlings combined, populations are plotted using scores calculated from median loadings, with adults as orange circles and seedlings as teal triangles. DA_pit_ was omitted from the combined adult and seedling PCA due to methodological differences in adults vs seedlings. Before estimating loadings, LA, SLA, d_h_, VD, and DA_pit_ were log‐transformed, and all traits were standardized. DA_pit_, pit diameter aperture; d_h_, hydraulically weighted vessel diameter; LA, leaf area; LDMC, leaf dry matter content; SLA, specific leaf area; TFW, thickness of fiber wall; VD, vessel density; WSG, wood specific gravity.

For adults, the first two dimensions of functional trait space represent a hydraulic safety–efficiency axis (component 1), which explains a median of 33.5% of variance (95% CI: [30.0%, 37.2%]) and a tissue investment axis (component 2), which explains a median of 25.3% of variance (95% CI: [23.1%, 28.0%]; Fig. 3a). On one end of the hydraulic axis are ‘safe’ species with a high density of narrow vessels, small pits, dense wood, and thick fiber walls. On the other end are hydraulically ‘efficient’ species with a low density of wide vessels, large pits, less dense wood, and thin fiber walls. The tissue investment axis represents a spectrum from ‘cheap’ tissue (high SLA, low LDMC, low WSG) to ‘costly’ tissue (low SLA, high LDMC, high WSG).

Seedlings also exhibited the same tissue investment axis consisting of SLA, LDMC, and WSG (component 1, 32.6% [25.5%, 40.8%] of variance; Fig. 3b). However, the hydraulic safety–efficiency trade‐off was only represented by a negative relationship between d_h_ and VD (component 2, 24.9% [20.5%, 32.1%]). Unlike in adults, DA_pit_ was aligned with the tissue investment axis in seedlings, and WSG was only aligned with the tissue investment axis rather than both axes. When combining trait data from both seedlings and adults, populations of both stages could be plotted along the same two orthogonal functional axes described above, specifically the hydraulic axis represented by d_h_ and VD (component 1, 29.6% [26.7%, 32.7%]) and the tissue investment axis represented by SLA and LDMC (component 2, 29.2% [26.8%, 31.9%]; Fig. 3c). DA_pit_ was not included in the combined seedling and adult dataset because the differences in seedling and adult values may be due to methodology (see the Materials and Methods section). Full loadings are provided in Table S2.

**Fig. 4:**
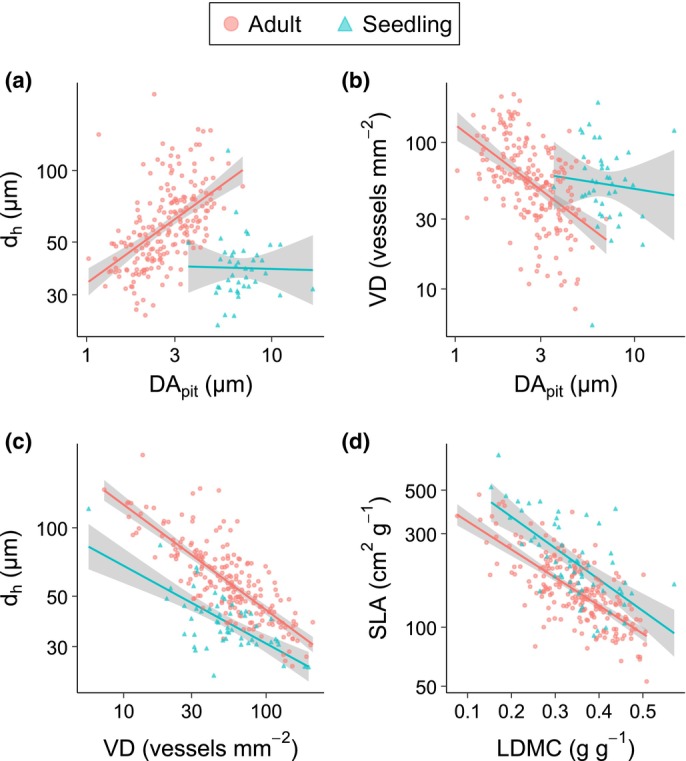
Trait correlations. (a) DA_pit_ and d_h_ are positively correlated for adults (ρ = 0.52) but not for seedlings (ρ = −0.02). (b) DA_pit_ and VD are negatively correlated for adults (ρ = −0.50) but not for seedlings (ρ = −0.08). (c) VD and d_h_ are negatively correlated for both adults (ρ = −0.78) and seedlings (ρ = −0.68), but seedlings have a less negative slope and a lower intercept compared to adults. (d) LDMC and SLA are negatively correlated for both adults (ρ = −0.71) and seedlings (ρ = −0.65), and the difference in slopes between seedlings and adults is not significant, though the intercept for seedlings is higher than for adults. Results are significant when the 95% credible interval does not include zero. Adults are plotted as orange circles, and seedlings are plotted as teal triangles. Note that all traits except LDMC are shown on log scales. DA_pit_, pit diameter aperture; d_h_, hydraulically weighted vessel diameter; LDMC, leaf dry matter content; SLA, specific leaf area; VD, vessel density.

The hydraulic safety–efficiency axes differed between adults and seedlings because correlations among wood traits differed between the two stages. DA_pit_ was correlated with d_h_ in adults (mean ρ = 0.52, 95% CI: [0.41, 0.61]) but not in seedlings (ρ = −0.02, [−0.32, 0.28]; Fig. 4a). Similarly, DA_pit_ was correlated with VD in adults (ρ = −0.50, [−0.60, −0.39]) but not in seedlings (ρ = −0.08, [−0.36, 0.22]; Fig. 4b). These results explain why DA_pit_ was aligned with these traits in the adult PCA but not in the seedling PCA. VD and d_h_ were negatively correlated in both adults (ρ = −0.78, [−0.83, −0.73]) and seedlings (ρ = −0.68, [−0.81, −0.50]; Fig. 4c), yet the slope of this relationship was slightly shallower in seedlings (β_seedling_ – β_adult_ = 0.19 [0.01, 0.38]), and the intercept was much lower for seedlings (α_seedling_ – α_adult_ = −1.03 [−1.20, −0.85], using standardized trait values), representing a different relationship between d_h_ and VD in seedlings than compared to adults. This means that for a given VD seedlings have narrower vessels (d_h_) than adults, and this gap is even greater when VD is low. SLA and LDMC were also negatively correlated in both adults (ρ = −0.71, [−0.77, −0.64]) and seedlings (ρ = −0.65, [−0.79, −0.49]; Fig. 4d). The slope of this relationship did not differ between seedlings and adults (β_seedling_ – β_adult_ = −0.08 [−0.27, 0.12]), but the intercept was higher for seedlings (α_seedling_ – α_adult_ = 0.75 [0.56, 0.95]), meaning that seedlings tend to have slightly higher SLA than adults for a given LDMC value.

## Discussion

### Ontogenetic trait shifts

In line with our predictions, d_h_ increased from the seedling to the adult stage (Fig. 1). Small seedlings with narrow and short stems have biophysical or developmental constraints that limit their ability to develop large vessels, and the correlation between plant height and vessel diameter has been widely observed (Hietz *et al*., 2017; Olson *et al*., 2018). Since d_h_ and VD are negatively correlated among adult trees in tropical dry forests (González‐M. *et al*., 2021), we predicted that VD would decrease in tandem with the increase in d_h_. Instead, VD did not exhibit ontogenetic shifts. Rather than shifting along a d_h_–VD axis, trees increased d_h_ as they grew without changing VD. In our study, d_h_ and VD were negatively correlated in both seedlings and adults, but seedlings had smaller vessels for a given VD (Fig. 4), meaning that seedlings had lower vessel lumen fractions given VD. Lower vessel lumen fraction is associated with lower risk of embolism (Avila *et al*., 2023), but seedlings are already less vulnerable to embolism due to their short height (Olson *et al*., 2014, 2018). All else equal, lower vessel lumen fraction also reduces conductivity (Zanne *et al*., 2010), so by having fewer vessels for a given vessel size, seedlings are not maximizing conductivity to the extent that adults do.

We observed a steep decrease in DA_pit_ between seedlings and adults (Fig. 1), though this may be due to differences in methodology. Our seedling samples were kept hydrated, but our adult samples were dried and then re‐hydrated, and dehydration has been shown to irreversibly reduce pit membrane thickness (Li *et al*., 2016; Zhang *et al*., 2017; Kotowska *et al*., 2020). Therefore, we attribute the observed ontogenetic differences in DA_pit_ to methodological factors.

As predicted, we observed increasing LDMC and decreasing SLA (Fig. 1), reflecting a shift of increasing tissue investment from seedlings to adults. This increase in tissue investment is related to the shift along the leaf economic spectrum that has been widely observed in many species and environments (Fortunel *et al.*, 2020; Barton, 2024). Also as predicted, WSG increased over ontogeny, likely reflecting a similar shift from fast‐growing acquisitive seedlings to slow‐growing conservative adults. Increasing WSG could be related to an increasing need for mechanical support, which is why we predicted that TFW would increase, but TFW did not significantly change from seedlings to adults. Fiber wall thickness is a component of wood density, but wood density is influenced by a complex set of traits, and species with the same wood density may have very different fiber wall fractions (Ziemińska *et al*., 2013). Thus, ontogenetic shifts in WSG are not necessarily linked to shifts in TFW.

### Ontogenetic shifts across a rainfall gradient

While the magnitude of trait shifts differed among certain pairs of sites and for certain traits, there were few consistent patterns along the rainfall gradient (Fig. 2). Contrary to our predictions, wetter sites did not have stronger ontogenetic shifts for any traits, except perhaps for LA. By contrast, ontogenetic shifts in SLA appeared to be greater in drier sites. The magnitude of shifts in d_h_ did not differ among any sites despite the gradient of water availability. Therefore, the shifts in d_h_ that we observed are likely due to ontogenetic differences in height, which strongly predicts vessel diameter across plants (Olson *et al*., 2018), and not due to ontogenetic differences in water environments (Fajardo *et al*., 2020). Our rainfall gradient consisted of four sites, but more research at additional sites is needed to study the role of environmental conditions in determining ontogenetic trait shifts.

In addition to differences among sites, leaf phenology and growth form explained some of the variation in the magnitude of ontogenetic shifts among species (Fig. S2). For example, SLA decreased more steeply for deciduous species than for evergreen species. While deciduous species tend to have higher SLA than evergreen species both as seedlings (Poorter & Markesteijn, 2008) and as adults (Vargas *et al*., 2021), we found that the gap in SLA was wider as seedlings. As species grew to adults, the SLA of deciduous species decreased faster, narrowing the gap in SLA between deciduous and evergreen adults (Fig. S3). To our knowledge, this is the first time that these differences in SLA over ontogeny have been reported for deciduous and nondeciduous species. Although not statistically significant, we also observed that vessel diameter tended to increase more steeply from the seedling to adult stage for lianas than for free‐standing species. Liana seedlings, which are mostly self‐supporting, had similar d_h_ values to tree or shrub seedlings (Puentes‐Marín *et al*., 2024), but as they matured, adult lianas had substantially higher d_h_ values than free‐standing adults in our dataset (Fig. S3). Ontogenetic shifts in hydraulic traits may differ between lianas and trees due to the transition lianas undergo from self‐supporting seedlings to non‐self‐supporting adults. Overall, the relative lack of differences across our rainfall gradient, combined with the differences we observed based on leaf phenology and growth form, suggests that developmental constraints, rather than changing environmental conditions, may be the most important factor in driving ontogenetic trait shifts. Much of the variation in ontogenetic trait shifts remains unexplained. Further research should consider the interaction between species and ontogeny to understand the variation in ontogenetic trait shifts.

### Coordination of functional traits for adults and seedlings

For both adults and seedlings, d_h_ and VD formed a hydraulic safety–efficiency axis orthogonal to the tissue investment axis (Fig. 3), but the hydraulic safety–efficiency axis differed between stages in several ways. Unlike in adults, WSG and DA_pit_ were not associated with this safety–efficiency axis in seedlings. DA_pit_ was strongly correlated with both d_h_ and VD for adults but was not correlated with either for seedlings (Fig. 4). DA_pit_ is associated with drought‐induced risk of embolism in adults (Choat *et al*., 2008; Levionnois *et al*., 2021), but seedlings may be exposed to fewer embolisms due to their smaller stature and lower tension in the water column. Seedlings also have narrower vessels due to developmental constraints, and this may also reduce the risk of embolism for seedlings, though d_h_ is not necessarily linked to embolism risk (Hacke *et al*., 2017; Lens *et al*., 2022). If seedlings face less pressure to maximize safety, DA_pit_ may be decoupled from other wood traits associated with the safety–efficiency axis. Seedlings do exhibit a hydraulic safety–efficiency axis characterized by d_h_ and VD, and these traits were negatively correlated for both seedlings and adults (Fig. 4). However, seedlings had much lower d_h_ for a given value of VD, and the slope of the relationship between d_h_ and VD was lower. If the d_h_–VD axis in adults represents a trade‐off between hydraulic safety and hydraulic efficiency, then seedlings are failing to maximize this trade‐off. That is, compared to adults, seedlings appear to be less safe for a given level of efficiency and less efficient for a given level of safety.

Adults exhibit the strong trade‐off in hydraulic safety vs hydraulic efficiency that has been reported in dry forests, but this trade‐off appears weaker in seedlings. Plants in dry and highly seasonal environments must strike a balance between maximizing safety and efficiency, creating a trade‐off, but plants in wetter and less seasonal environments often have both low safety and low efficiency (Liu *et al*., 2021). The weaker trade‐off we observe in seedlings suggests that seedlings may similarly be failing to maximize safety and efficiency. As trees grow and the risk of embolism increases, though, the pressure to maximize hydraulic safety and efficiency may increase, resulting in a strong trade‐off for adults in tropical dry forests. Even if the safety–efficiency trade‐off is weaker for seedlings, seedlings face a much higher risk of mortality than adults, and seedling survival and establishment in tropical dry forests are dependent on water availability (Bhadouria *et al*., 2016), so drought is still a strong selective pressure that should influence seedling traits. Seedlings may rely on different strategies than adults to deal with drought. For seedlings, whose shallow roots cannot access deeper water resources, drought survival may depend on the reservoir of water, nutrients, and carbohydrates stored in parenchyma. In relatively arid dry forest sites, seedlings with higher parenchyma fractions in their xylem had higher survival, whereas vessel traits did not predict survival (González‐Melo *et al*., 2025). In this way, the traits that enable seedlings to survive drought may not fall along the hydraulic safety–efficiency continuum seen in adults. Our results suggest that vessel traits are perhaps less important for determining seedling performance than for adults in tropical dry forests, although vessel traits do weakly predict growth in dry forest seedlings (González‐Melo *et al*., 2025). More research is needed to determine whether vessel traits explain seedling growth, survival, and abundance and to determine whether wood anatomy traits impact outcomes in similar ways between seedlings and adults.

Seedlings and adults both exhibited the same tissue investment axis, as predicted, with ‘cheap’ tissues characterized by high SLA, low LDMC, and high WSG and with ‘costly’ tissues characterized by low SLA, high LDMC, and high WSG (Fig. 3). SLA and LDMC were negatively correlated for both seedlings and adults, and the slope of the relationship was similar between both stages (Fig. 4). Compared to adults, seedlings tended to have slightly higher SLA for a given value of LDMC, though the general relationship between SLA and LDMC appears to be quite similar between seedlings and adults, suggesting that seedlings and adults share a single tissue investment axis.

### Conclusions

Our results are in line with the acquisitive‐to‐conservative ontogenetic shifts that have been observed in other species and ecosystems (Plourde *et al*., 2015; Hietz *et al*., 2017; Dayrell *et al*., 2018; Barton, 2024), but we also bring attention to ontogenetic changes in other functions, such as water transport, which are also crucial for plants. Rather than shifting along a single hydraulic trait axis, the trade‐offs among wood anatomy traits changed over ontogeny. Ontogenetic shifts in wood anatomy traits are more complex than shifts in leaf traits due to the multiple functions of wood and the combined roles of environment and endogenous factors (e.g. changes in age or size). Unlike leaf economics traits, many wood traits are strongly constrained by plant size (Olson *et al*., 2014; Fajardo *et al*., 2020). As trees grow, they need to invest more energy and resources in building and maintaining support tissues which can accentuate the functional trade‐offs involving wood anatomical traits (Givnish, 1995). The result is that biophysical and biomechanical constraints may become more pronounced with size, leading to stronger and more visible trade‐offs for adults than for seedlings.

In tropical dry forests, both seedlings and adults must cope with drought, but our results show that traits related to hydraulic function are integrated differently in these two stages. While wood traits in adult trees in dry forests are constrained by the trade‐off between hydraulic safety and hydraulic efficiency, seedlings, which are at lower risk of embolism, are less constrained by this particular trade‐off. As forests around the world face increasing drought intensity (Brodribb *et al*., 2020; Hartmann *et al*., 2022), understanding trait shifts and stage‐specific trade‐offs is important for predicting responses of seedlings and adult trees under these new conditions.

## Competing interests

None declared.

## Author contributions

The study was designed by PJW, EFZ, and MNU. Seedling data were collected by AG‐M, BS‐N, JPB‐T, JMC, ÁI‐P, EM, JPM, NP, KR, FRB, JFS, and MNU. Fieldwork and logistics for adult data were conducted by BS‐N, RG‐M, NN, JAF, DG‐V, FG, ÁI‐P, RL‐C, JN, CP, and VS. Data analyses were performed by PJW with contributions from MNU and EFZ. The first drafts of the manuscript were written by PJW with important inputs from EFZ, AG‐M, BS‐N, RG‐M, NN, and MNU. All authors contributed to revising and rewriting the manuscript.

## Disclaimer

The New Phytologist Foundation remains neutral with regard to jurisdictional claims in maps and in any institutional affiliations.

## Supporting information


**Fig. S1** Differences in ontogenetic trait shifts across sites, without including effects of phenology or growth form.
**Fig. S2** Differences in ontogenetic trait shifts between leaf phenologies and growth forms, after accounting for differences among sites.
**Fig. S3** Trait values and ontogenetic shifts by leaf phenology and growth form.
**Methods S1** Bayesian PCA.
**Table S1** Summary of trait values for adults and seedlings.
**Table S2** Loadings for functional trait PCAs.
Please note: Wiley is not responsible for the content or functionality of any Supporting Information supplied by the authors. Any queries (other than missing material) should be directed to the *New Phytologist* Central Office.

## Data Availability

The data used in this study are available in the Deep Blue Data repository at https://doi.org/10.7302/kjr3‐d129. The code used for analyses is available at https://github.com/pwilliams0/TDF_ontogeny.
