# Supplementary material for: Ontogenetic shifts in wood anatomy and leaf traits in tropical dry forests
Source: New Phytol. 2025 Nov 10;249(2):848–59. doi: 10.1111/nph.70725 (PMC12712423; doi:10.1111/nph.70725)
Supplement: Supplementary file 1 — Fig. S1 Differences in ontogenetic trait shifts across sites, without including effects of phenology or growth form. Fig. S2 Differences in ontogenetic trait shifts between leaf phenologies and growth forms, after accounting for differences among sites. Fig. S3 Trait values and ontogenetic shifts by leaf phenology and growth form. Methods S1 Bayesian PCA. Table S1 Summary of trait values for adults and seedlings. Table S2 Loadings for functional trait PCAs. Please note: Wiley is not responsible for the content or functionality of any Supporting Information supplied by the authors. Any queries (other than missing material) should be directed to the New Phytologist Central Office. [file NPH-249-848-s001.pdf]

NEW PHYTOLOGIST SUPPORTING INFORMATION

Title: Ontogenetic shifts in wood anatomy and leaf traits in tropical dry forests

Authors: Peter J. Williams, Elise F. Zipkin, Andrés González-Melo, Beatriz Salgado-Negret, Roy González-M., Natalia Norden, Juan Pablo Benavides-Tocarruncho, Juan Manuel Cely, Julio Abad Ferrer, Daniel García-Villalobos, Fabián Garzón, Álvaro Idárraga-Piedrahita, René López-Camacho, Esteban Moreno, Jhon Nieto, Camila Pizano, Juliana Puentes-Marín, Nancy Pulido, Katherine Rivera, Felipe Rojas-Bautista, Viviana Salinas, Juan Felipe Solorzano, & María Natalia Umaña

Article acceptance date: 18 October 2025

Methods S1:

Bayesian PCA

We first calculated the covariance matrix for all traits by assuming that the standardized trait values were derived from a multinormal distribution. We then calculated the loadings and applied a varimax rotation to the first two components of the loadings matrix. While PCA maximizes the variance explained by each component, varimax rotation ensures that traits are strongly aligned with the components. In our case, rotation makes components more interpretable, since each component is correlated with a set of traits, reflecting underlying and orthogonal functional axes.

Each posterior draw of our Bayesian PCA model resulted in a varimax-rotated loadings matrix. However, the signs of the loadings matrices are arbitrary, so the posterior distribution of loadings was often bimodal. Furthermore, while some traits had consistently high loading values for a single axis, slight changes in the variance explained by each axis could affect whether that axis was assigned as component 1 or component 2. To address these issues, we transformed the loadings matrices to ensure consistent interpretability across all posterior draws. We first identified the trait that had the highest median loading for a given component. For example, before transforming the loadings matrices, d_h is the trait most strongly associated with component 1 for adults. Then, for each posterior loadings matrix, we defined component 1 as the component for which the most strongly associated trait has the highest loading. For adults, the component for which d_h was most highly loaded became component 1, regardless of which component explained the highest variance, to maintain interpretability of trait loadings. Then, for each component and each posterior loadings matrix, we fixed the sign of the most strongly associated trait as positive and adjusted the signs of all other traits accordingly. So, for example, if the loading of d_h for component 1 in a given loadings matrix was positive, we would not change any of the component 1 loadings, but if the loading of d_h were negative, we would flip the signs of all loadings for component 1. With the final set of loadings matrices, we determined the median and 95% credible intervals for the loadings of each trait and component, and we determined the median and 95% CIs for the variance explained by each component. We also calculated the scores for each population and the variance explained by each component when using median loading values.

Table S1: Summary of traits for adults and seedlings, including median values, minimum values (Min.), first quartile (Q1), third quartile (Q3), maximum values (Max.), and sample size (N). Mean values are not shown because many traits are lognormally distributed. Note that traits vary in the number of populations sampled.

**Trait (abbreviation, units)**		**Median**	**Min.**	**Q1**	**Q3**	**Max.**	**N**
Leaf area (LA, cm^2)	*Adults*	54.6	4.0	28.3	114.9	788.0	212
	Seedlings	40.5	4.8	18.5	62.6	205.8	57
Leaf dry matter content (LDMC, $g\ g^{-1}$)	*Adults*	0.357	0.076	0.289	0.403	0.509	214
	Seedlings	0.349	0.154	0.299	0.397	0.572	57
Specific leaf area (SLA, $cm^2\ g^{-1}$)	*Adults*	150.2	52.9	117.3	198.9	474.2	212
	Seedlings	207.9	90.7	154.8	323.8	755.3	57
Wood specific gravity (WSG)	*Adults*	0.631	0.238	0.511	0.719	0.879	219
	Seedlings	0.607	0.238	0.528	0.654	0.793	57
Thickness of fiber wall (TFW, µm)	*Adults*	5.18	1.68	4.49	6.21	9.66	203
	Seedlings	3.82	2.39	3.14	4.97	9.51	52
Hydraulically weighted vessel diameter (d_h, µm)	*Adults*	56.9	24.6	44.5	74.6	209.1	203
	Seedlings	37.7	22.4	31.9	44.4	121.3	52
Vessel density (VD, vessels mm^{-2})	*Adults*	56.2	7.3	34.7	81.5	212.9	203
	Seedlings	50.4	5.7	32.8	72.9	198.8	52
Pit diameter aperture (DA_{pit}, µm)	*Adults*	2.73	1.03	2.04	3.49	6.97	203
	Seedlings	6.58	3.55	5.82	7.51	16.67	41

Table S2: Median loadings with 95% credible intervals for the functional trait PCAs shown in Fig. **3**. Loadings > 0.5 or < −0.5 are bold. LA, leaf area; LDMC, leaf dry matter content; SLA, specific leaf area; WSG, wood specific gravity; TFW, thickness of fiber wall; d_h, hydraulically weighted vessel diameter; VD, vessel density; DA_{pit}, pit diameter aperture.

**Trait**	**Adults**		**Seedlings**		**Adults & Seedlings**	
	Component 1	Component 2	Component 1	Component 2	Component 1	Component 2
LA	0.50<br>[0.32, 0.62]	0.14<br>[−0.06, 0.39]	0.40<br>[−0.11, 0.73]	0.11<br>[−0.55, 0.65]	**0.52**<br>[0.31, 0.64]	0.29<br>[0.09, 0.51]
LDMC	0.00<br>[−0.19, 0.12]	**0.91**<br>[0.88, 0.93]	**0.82**<br>[0.60, 0.92]	0.00<br>[−0.42, 0.42]	0.01<br>[−0.18, 0.15]	**0.89**<br>[0.84, 0.92]
SLA	−0.06<br>[−0.17, 0.11]	**−0.87**<br>[−0.90, −0.82]	**−0.81**<br>[−0.90, −0.34]	0.17<br>[−0.68, 0.72]	−0.10<br>[−0.26, 0.07]	**−0.85**<br>[−0.89, −0.80]
WSG	**−0.53**<br>[−0.68, −0.35]	**0.54**<br>[0.29, 0.69]	**0.79**<br>[0.50, 0.88]	−0.08<br>[−0.52, 0.38]	−0.50<br>[−0.66, −0.30]	**0.63**<br>[0.44, 0.75]
TFW	−0.47<br>[−0.60, −0.27]	−0.23<br>[−0.48, 0.01]	0.42<br>[−0.22, 0.75]	0.28<br>[−0.61, 0.83]	−0.29<br>[−0.48, −0.03]	0.10<br>[−0.12, 0.35]
d_h	**0.87**<br>[0.82, 0.91]	−0.19<br>[−0.33, −0.01]	−0.04<br>[−0.79, 0.27]	**0.87**<br>[0.11, 0.95]	**0.87**<br>[0.82, 0.90]	−0.06<br>[−0.20, 0.15]
VD	**−0.84**<br>[−0.88, −0.79]	0.01<br>[−0.15, 0.16]	0.14<br>[−0.19, 0.75]	**−0.85**<br>[−0.94, 0.14]	**−0.83**<br>[−0.87, −0.78]	0.06<br>[−0.10, 0.22]
DA_{pit}	**0.68**<br>[0.58, 0.76]	−0.09<br>[−0.26, 0.06]	−0.41<br>[−0.75, 0.18]	0.10<br>[−0.80, 0.77]	NA	NA

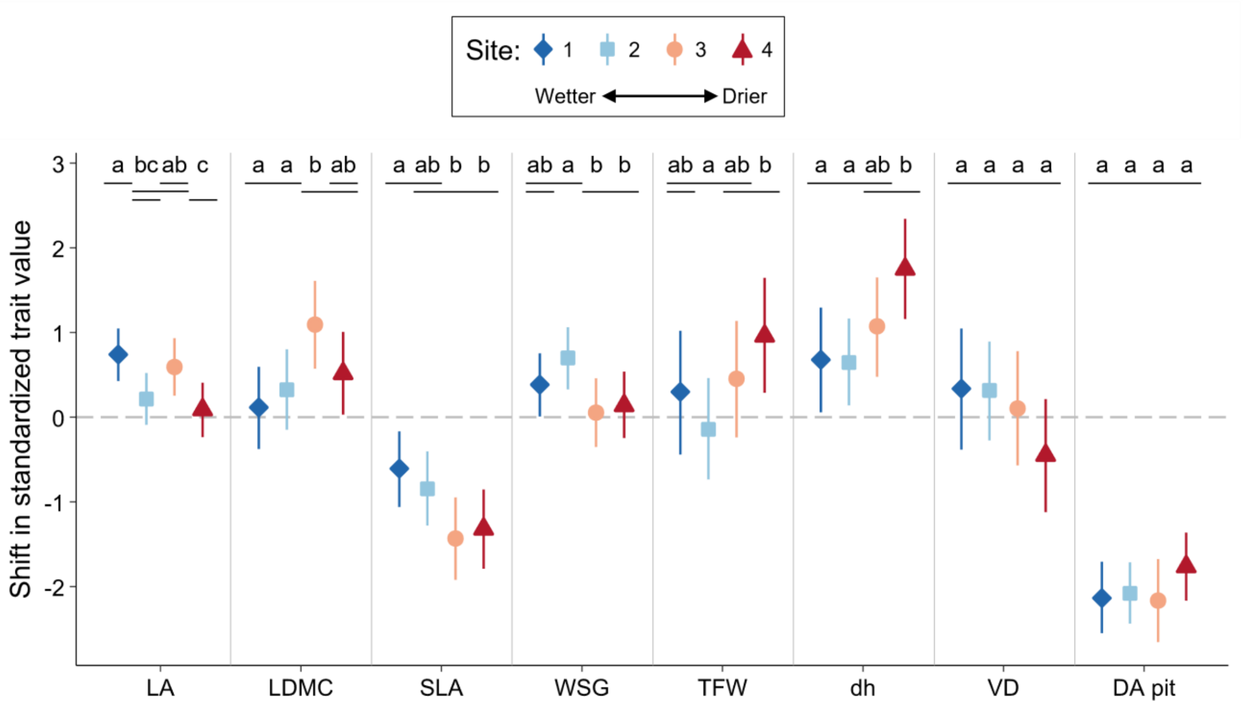

Fig. S1: Differences in ontogenetic trait shifts across sites, without including effects of phenology or growth form. Positive values indicate an increase in trait value from the seedling stage to the adult stage, while negative values indicate a decrease in trait value. Colored shapes represent mean estimated trait shifts for a given site, and colored lines represent 95% credible intervals. For a given trait, sites with significantly different trait shifts are indicated by different letters and non-overlapping lines at the top of the figure. See Table 1 for site information. LA, leaf area; LDMC, leaf dry matter content; SLA, specific leaf area; WSG, wood specific gravity; TFW, thickness of fiber wall; d_h, hydraulically weighted vessel diameter; VD, vessel density; DA_{pit}, pit diameter aperture.

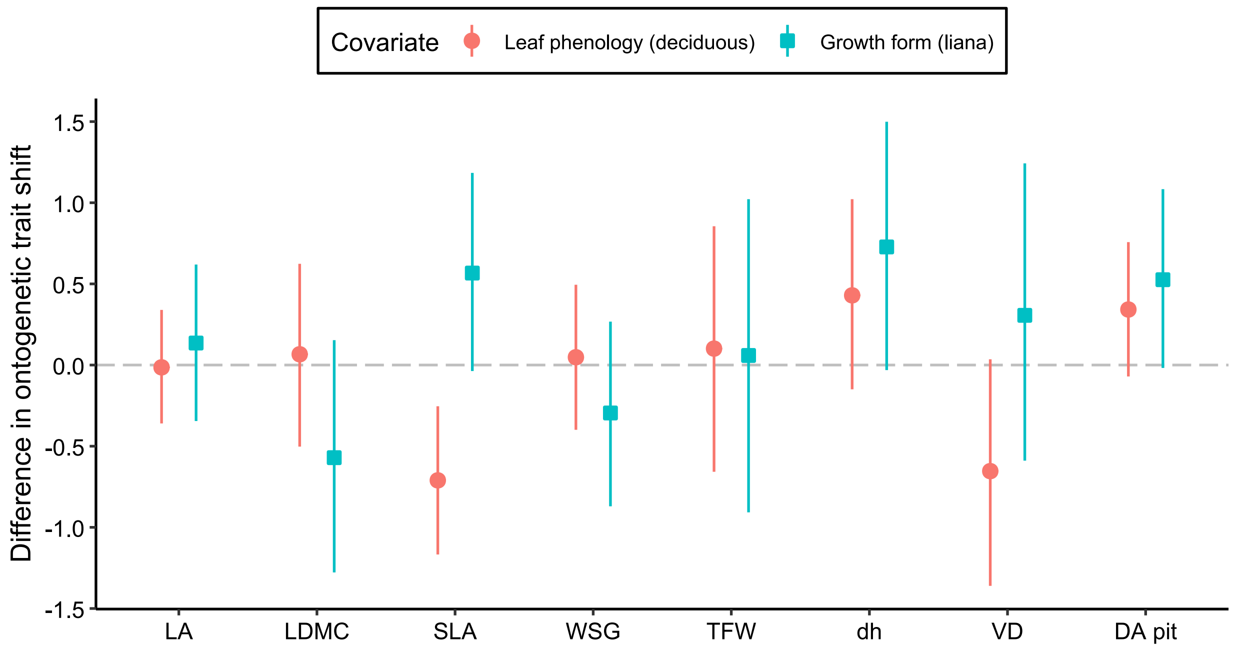

Fig. S2: Differences in ontogenetic trait shifts between leaf phenologies and growth forms, after accounting for differences among sites. Positive values indicate a more positive (or less negative) shift from the seedling stage to the adult stage among deciduous species compared to evergreen species, or among lianas compared to free-standing species, while negative values indicate a more negative (or less positive) ontogenetic shift. For example, SLA decreased from the seedling to adult stage overall, but SLA decreased more steeply for deciduous species than for evergreen species, while SLA tended to decrease less steeply for lianas than for free-standing species. As another example, d_h tended to increase from the seedling to adult stage overall, but d_h tended to increase more steeply lianas than for free-standing species. See Fig. 1 for overall ontogenetic shifts across all species. Colored shapes represent mean estimated trait shifts for a given group, and colored lines represent 95% credible intervals. LA, leaf area; LDMC, leaf dry matter content; SLA, specific leaf area; WSG, wood specific gravity; TFW, thickness of fiber wall; d_h, hydraulically weighted vessel diameter; VD, vessel density; DA_{pit}, pit diameter aperture.

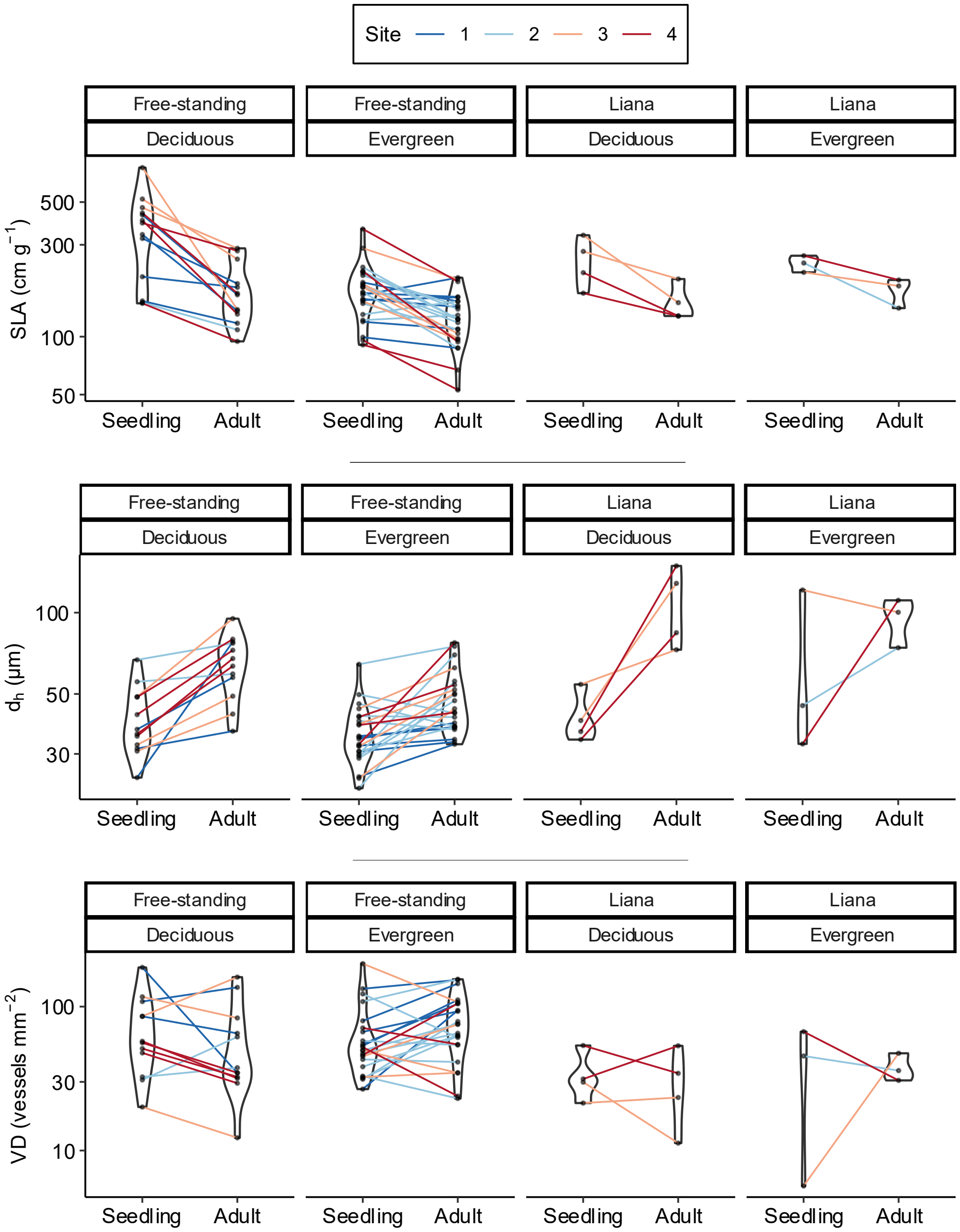

Fig. S3: Trait values and ontogenetic shifts by leaf phenology (deciduous vs. evergreen) and growth form (liana vs. free-standing). Deciduous species have a steeper decline in SLA (specific leaf area) from seedling to adult compared to evergreen species, and lianas tend to have shallower declines in SLA compared to free-standing species (top row). Lianas tend to have a steep increase in d_h (hydraulically weighted vessel diameter) compared to free-standing species (middle row). Deciduous species tend to have decreasing VD (vessel density) while evergreen species tend to have increasing VD (bottom row). Trait shifts are colored by site. Y axes are on log scales. Trait values plotted here are for the same species used to plot Figs. 1, 2, S1, and S2.
